# Investigating Software Requirements for Systems Supporting Task-Shifted Interventions: Usability Study

**DOI:** 10.2196/11346

**Published:** 2019-11-12

**Authors:** Pepijn Van de Ven, Ricardo Araya, Maria Clara P de Paula Couto, Maiara Garcia Henrique, Damien Meere, Ana Vilela Mendes, Tim J Peters, Antônio Seabra, Renato M Franzin, Paula Carvalho Pereda, Marcia Scazufca

**Affiliations:** 1 Health Research Institute, HIST Cluster University of Limerick Limerick Ireland; 2 King’s College London United Kingdom; 3 Friedrich-Schiller University Jena Germany; 4 Universidade de São Paulo São Paulo Brazil; 5 BT Ireland Limerick Ireland; 6 University of Bristol Bristol United Kingdom

**Keywords:** task-shifting, community health workers, depression, medical informatics

## Abstract

**Background:**

There is a considerable shortfall in specialized health care professionals worldwide to deliver health services, and this shortfall is especially pronounced in low-middle-income countries. This has led to the implementation of task-shifted interventions, in which specific tasks are moved away from highly qualified health workers to health workers with less training. The World Health Organization (WHO) has published recommendations for such interventions, but guidelines for software and systems supporting such interventions are not included.

**Objective:**

The objective of this study was to formulate a number of software requirements for computer systems supporting task-shifted interventions. As the treatment of mental health problems is generally considered to be a task for highly trained health care professionals, it poses interesting case studies for task-shifted interventions. Therefore, we illustrated the use of the identified software requirements in a mobile system created for a task-shifted depression intervention to be provided to older adults in deprived areas of São Paulo, Brazil.

**Methods:**

Using a set of recommendations based on the WHO’s guidance documentation for task-shifted interventions, we identified 9 software requirements that aim to support health workers in management and supervision, training, good relationship with other health workers, and community embeddedness of the intervention. These 9 software requirements were used to implement a system for the provision of a psychosocial depression intervention with mobile Android interfaces to structure interventions and collect data, and Web interfaces for supervision and support of the health care workers delivering the intervention. The system was tested in a 2-arm pilot study with 33 patients and 11 health workers. In all, 8 of these 11 health workers participated in a usability study subsequent to the pilot.

**Results:**

The qualitative and quantitative feedback obtained with the System Usability Scale suggest that the system was deemed to have a usability of between *OK* and *Good*. Nevertheless, some participants’ responses indicated that they felt they needed technical assistance to use the system. This was reinforced by answers obtained with perceived usefulness and ease of use questionnaires, which indicated some users felt that they had issues around correct use of the system and perceived ability to become skillful at using the system.

**Conclusions:**

Overall, these high-level requirements adequately captured the functionality required to enable the health workers to provide the intervention successfully. Nevertheless, the analysis of results indicated that some improvements were required for the system to be useable in a task-shifted intervention. The most important of these were better access to a training environment, access for supervisors to metadata such as duration of sessions or exercises to identify issues, and a more robust and human-error–proof approach to the availability of patient data on the mobile devices used during the intervention.

## Introduction

### Background

Health care systems worldwide, but especially those in low-middle-income countries (LMICs), struggle with the high demand for the specialized resources traditionally used in the delivery of health care interventions. To address the lack of specialized resources, the most common strategy followed has been that of task-shifting. This strategy involves moving specific tasks, where appropriate, from highly qualified health workers to health workers with less training and fewer qualifications, so as to make more efficient use of the available human resources [1]. Task-shifting has a long history in many guises with structured implementations in, for example, China and Thailand from the 1950s and 1970s onward, respectively [2,3]. In Africa, task-shifting has been used for various diseases and notably in response to the HIV/AIDS pandemic [4]. Task-shifting is not limited to LMICs. For example, Maier and Aiken found that of 39 countries covering Europe, the United States, Canada, Australia, and New Zealand, 27 countries made use of task-shifting from physicians to nurses [5]. In such settings, task-shifting may improve access to services or reduce their cost. In LMICs on the other hand, the use of task-shifting may have a more pronounced effect as it may allow the delivery of health services where this was not possible before because of the lack of human resources. Rather than shifting tasks to nurses, in LMICs, most of the task-shifted programs have been delivered by community health workers (CHWs) who are generally people of all ages, often community members, with no professional education but who receive a few months of training.

A review of task-shifting studies in LMICs found that task-shifting was a promising approach to efficiency improvements and the increased provision of services at a given quality and cost [6]. Notwithstanding the merits of this strategy, there have been problems with its implementation [6,7]. Between 1980 and 1990, many established task-shifted programs were discontinued because of poor implementation, resourcing issues, or the absence of lasting health outcome improvements [8]. In recognition of these and other challenges, the World Health Organization (WHO) collated a set of recommendations for successful implementation of task-shifting interventions [1]. Campbell and Scott [8] divided these recommendations into 5 categories and added a sixth as follows:

1. Strong management and supportive supervision
2. Appropriate selection of CHWs
3. Suitable training
4. Adequate retention and incentive structures for CHWs
5. Good relationship with other health care workers
6. Community embeddedness of personnel and intervention

Information and communications technology (ICT), though acknowledged as a useful tool in some task-shifting interventions [9], is overlooked in these recommendations as an opportunity to overcome some of the fundamental issues in supporting task-shifting. ICT can play an important role in the training, support, and supervision of CHWs delivering task-shifted interventions. The need for such functionality is illustrated by several studies of task-shifted interventions in mental health, which reported challenges around training and supervision of the intervention providers [10,11], treatment quality [12], and fidelity [13].

### Objectives

This study sought to address this gap by formulating a set of software requirements to extend the requirements listed by Campbell and Scott [8] and illustrating the use of these requirements in a software platform for the support of a task-shifted depression intervention. Depression is common among older adults [14-17], impacts negatively on quality of life [18,19], has negative social and health consequences [20-22], and increases health care utilization and costs [23,24]. There are effective treatments for depression in later life [25-27], but these are complex interventions, which often require specialized resources. One of the most salient barriers to deliver these programs is the lack of specialized staff [28-32]. Hence, a task-shifted depression intervention is a challenging, potentially impactful demonstrator for the task-shifting software requirements we identified in this study. The intervention aimed to allow CHWs, who normally provide basic services to elderly citizens within Brazil’s family unit-based health system [33], to deliver a complex psychosocial intervention to older adults residing in poor neighborhoods of São Paulo, Brazil. As far as we are aware, specific software requirements for ICT systems that play supporting roles in task-shifting interventions are not currently available. Although the app described focusses on mental health interventions, the applicability of the chosen approach is not limited to the domain of mental health.

## Methods

### Definition of Task-Shifting Software System Requirements

Four of the recommendation categories proposed by Campbell and Scott are relevant to the functionality that can be provided by ICT systems: management and supervision, training, good relationship with other health care workers, and community embeddedness. During the initial stages of the project, a needs analysis and *use case* scenarios were employed to define software requirements for these 4 categories. In this phase, a psychiatrist and 3 psychologists were involved in the drafting of a number of use cases to describe a typical use of the system and the functionality required to support the CHWs in delivering the intervention. Subsequently, a nonfunctional prototype based on mock-ups was tested and discussed further, resulting in updated use cases and the first version of the requirement specification. The first version of the requirement specification formed the basis of a functional prototypes system that was presented to CHWs. Their feedback was incorporated in the final pilot system.

#### Software Requirements for Strong Management and Supportive Supervision

The mobile nature of modern ICT systems allows users to manage their tasks effectively and obtain appropriate supervision input as and when required. Such interactions can be user initiated, prompted by the system, or may result from supervisor intervention. The developed system contributes to such management and supervision by implementing the requirements described further.

##### Requirement 1: Guidance

The ICT system should provide the CHW with a means of structuring the intervention, automatically presenting the key aspects of the intervention at the appropriate time.

##### Requirement 2: Decision Support

The system should either aid in decision making, or automatically make certain decisions for the care provider.

##### Requirement 3: Supervision

The system should enable supervisors of the CHWs to remotely monitor the intervention, assess progress and potential issues, and initiate corrective action when required.

##### Requirement 4: Accountability

The system should provide all users with the trust and confidence that (1) they receive automated prompts in regard to their duties; (2) the recording of the execution of such duties is performed automatically insofar as practically and ethically possible; (3) any deviation from these duties is flagged accordingly and reported to line managers; and (4) any storage and communication of patient data are performed in a secure manner.

##### Requirement 5: Record Keeping

The system should automatically maintain records of information resulting from the intervention. These records should then be available for later sessions to support Guidance and Decision Support and for Supervision and Accountability purposes.

#### Software Requirements for Suitable Training

Characteristics of learning identified as important for task-shifting [34], such as being learner-centered, experiential, problem-orientated, and context-appropriate can be appropriately provided by ICT, especially as a refresher after initial, more formal training.

##### Requirement 6: Training Environment

The system should allow CHWs to quickly review the content of a specific session and practice delivery of the intervention and all its exercises.

#### Software Requirements for Good Relationship With Other Health Care Workers

The relationship between CHWs within a team and between CHWs and their supervisors is crucial to a successful task-shifted intervention [35]. ICT can contribute to a good relationship by providing effective and transparent communication between CHWs among themselves, and between CHWs and their supervisors or general practitioners.

##### Requirement 7: Automated Communication

The system should automatically communicate important information and events to relevant stakeholders. It should be clear to all stakeholders what information is relayed to whom. Where required, stakeholders should be able to respond.

##### Requirement 8: User-Initiated Communication

The system should allow immediate communication with relevant stakeholders involved in the care of that particular patient.

#### Software Requirements for Community Embeddedness of Personnel and Intervention

Campbell and Scott [8] defined community embeddedness as “...when community members ‘own’ the project by having substantial control over the selection, monitoring, activities and priority-setting of CHWs.” Whereas this definition goes far beyond the physical location of the intervention, an important aspect of community embeddedness is that interventions can be provided where this is deemed most suitable by the community, and often this may be the patient’s own home. Especially for the older adults targeted in this work, mobility may be a limiting factor in the delivery of care through health centers.

##### Requirement 9: Delivery in the Community

It should be possible to deliver the full intervention in the community, certainly in a location acceptable to the patient and if necessary in their own home. Hence, all intervention resources and patient data should be accessible on a mobile device without direct connection to the internet, for instance through an app.

### Development of the Pilot System

The system requirements gathered in the early stages of this development process indicated the necessity for an architecture with interfaces for CHWs on mobile devices, a central storage of gathered data, and Web interfaces for supervisory actors. The resulting system, which was named PROACTIVE, contains a mobile Android app developed for tablets with a minimum screen size of 8″ and various interfaces accessible through a Web-based portal for remote supervision and management purposes.

#### The Psychosocial Intervention

One of the main goals of the intervention is to strengthen the autonomy of the patients and highlight the role they can play in their own therapy. The intervention combines psychosocial techniques tailored to individual participants with embedded support mechanisms for nonspecialist health workers delivering the intervention. Behavioral activation was used as the main psychosocial approach in view of its demonstrated feasibility and efficacy for the treatment of depression [36]. It is a simple technique to apply, requires only a short period of professional training [37], and is suitable for delivery by nonspecialists [38,39]. The intervention further incorporates elements of psychoeducation (education about depression and simple coping strategies) and relapse prevention.

#### Mobile Android Interfaces

**Figure 1:**
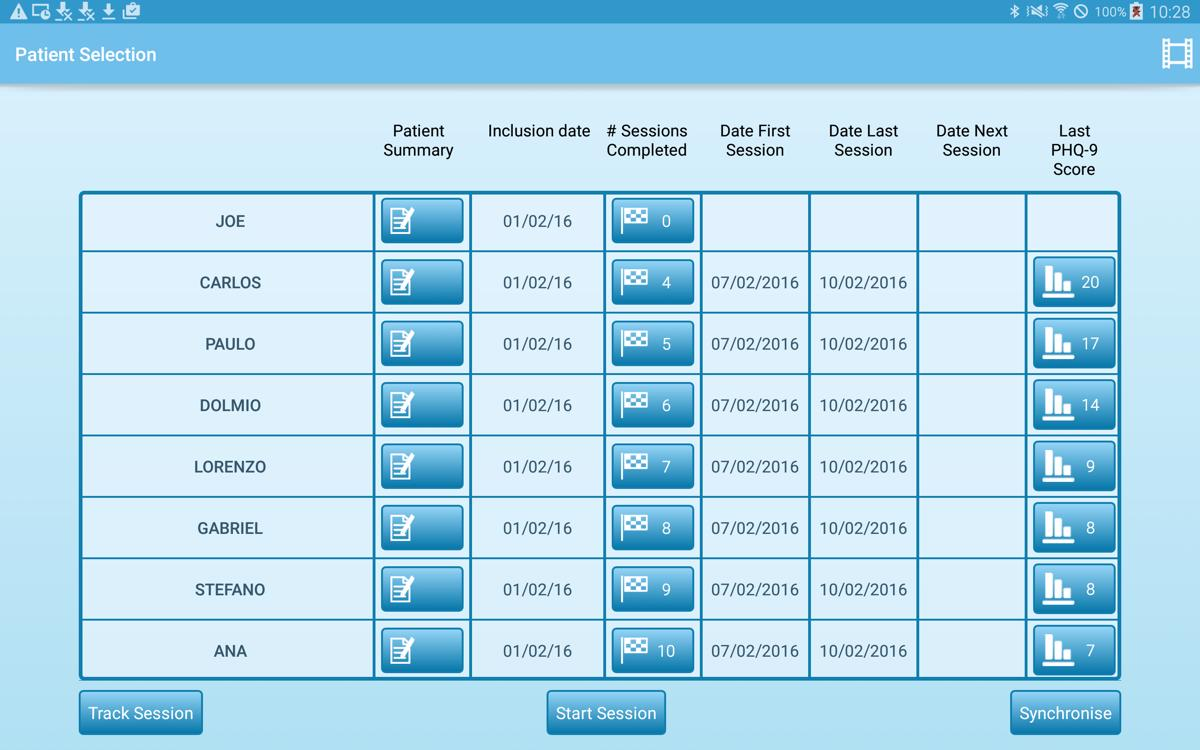
Patient overview screen.

The CHW performs all interactions and sessions with the patient using the Android app. Upon logging in with a username password combination, the Android app’s starting screen gives the user an overview of the patients in their care (see Figure 1).

When selecting a user and choosing *Start Session*, the correct session content will be compiled for the chosen user and an intervention session started. The various screens in each session can be navigated using swipe actions to move to neighboring screens similar to browsing through a book page by page. In addition, *chapters* in the session can be selected using a horizontal menu bar at the top of the screen, and pages within these chapters can be selected using a vertical menu at the left-hand side of the screen. Wherever the interface presents or requests dynamic patient or user-specific data, a custom-built application programming interface (API) is used to provide a quick and easy means of defining user interfaces with automated storage and retrieval of information. The information thus obtained is stored in database tables.

**Figure 2:**
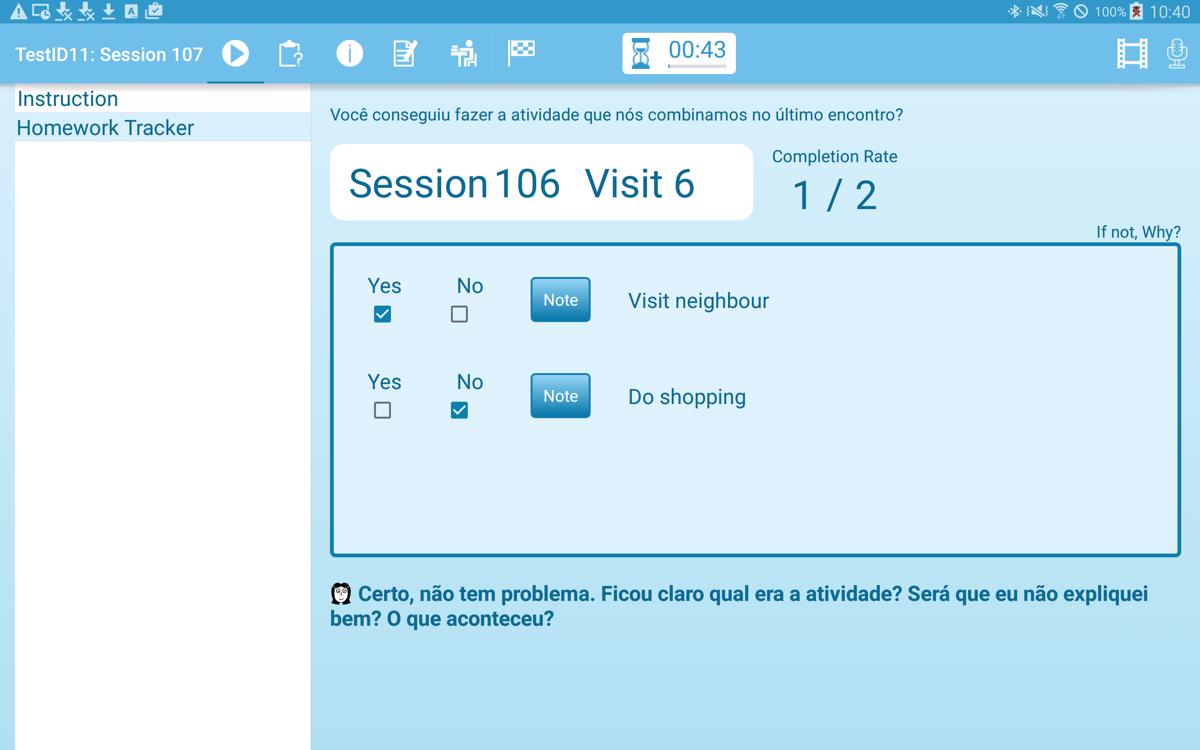
Homework review screen.

The first screen of every intervention session (apart from the first session) displays the homework assignments agreed upon in the previous session (Figure 2) and allows the patient and CHW to start the new session discussing and recording progress and exploring potential barriers and solutions for homework assignments.

**Figure 3:**
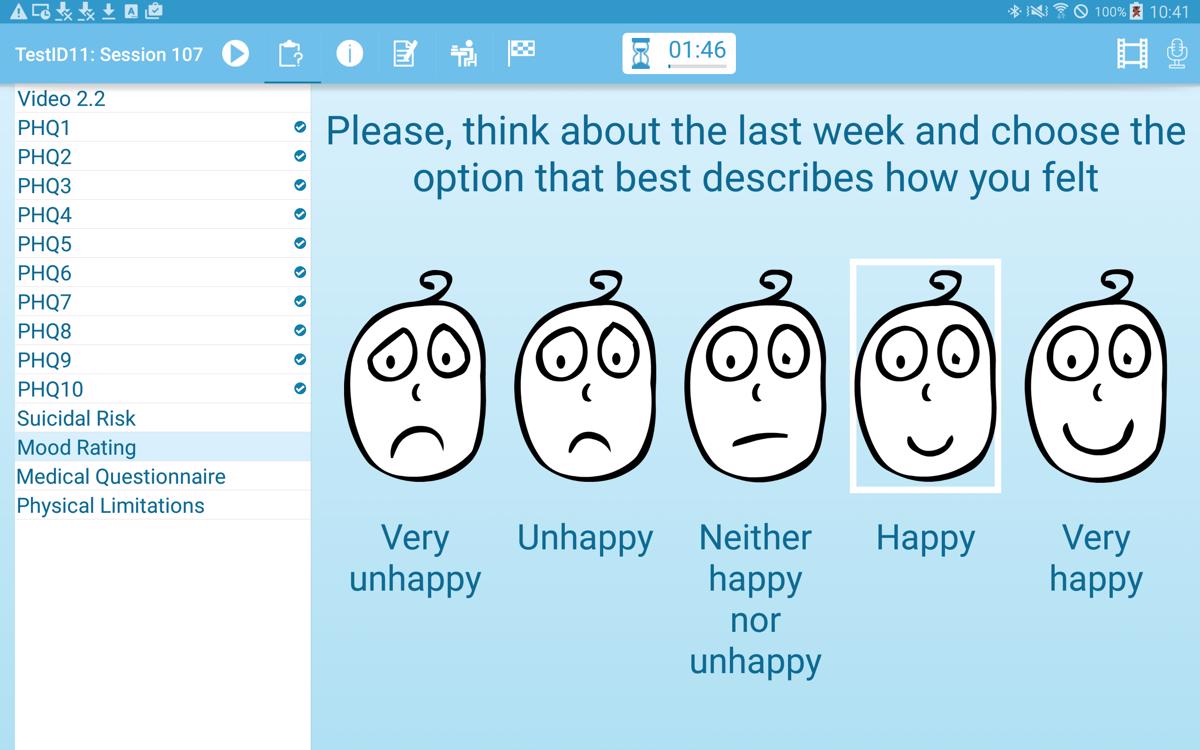
Mood rating screen.

**Figure 4:**
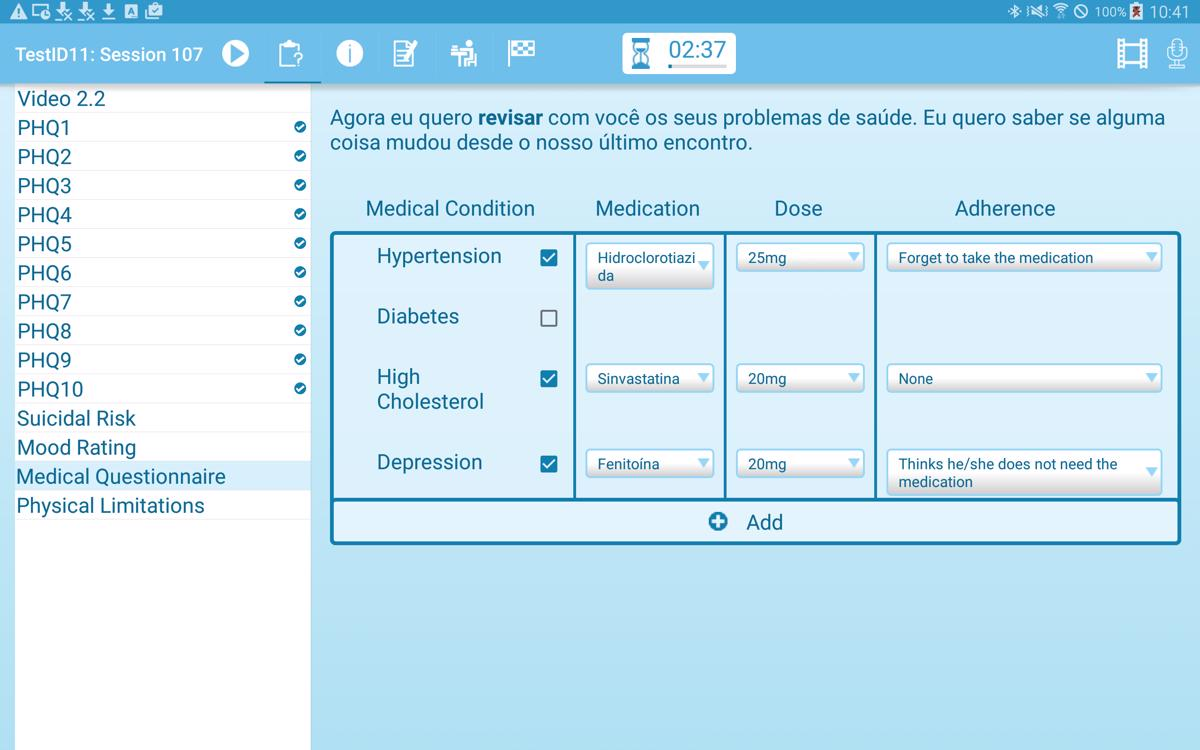
Medication questionnaire.

After reviewing their homework, the patients will respond to a number of questionnaires. On every visit, depression is assessed with the 9-item Patient Health Questionnaire (PHQ-9) [40], and mood is rated on a Likert rating scale (Figure 3). To assist CHWs in this task, a video presented during the first session explains to patients why and how the PHQ-9 is conducted, and the CHWs and patients have access to these videos throughout all future sessions. On the basis of the answers provided in the PHQ-9, the system will, if any of the answers indicate the presence of a depressive symptom, automatically add an extra question to assess how PHQ-9 symptoms have affected the patient’s life. Any positive response to the ninth question of the PHQ-9 on suicidal ideation automatically determines the immediate assessment of suicidal risk. For this, questions frequently used to assess immediate suicide risk (if there was any suicide attempt during the last 14 days and when was this attempt, and if the person has plans and means for another attempt) are shown on the screen. If the answer to any of these questions is positive, the CHW will immediately see instructions in the app on how to proceed in this situation, that is, to stay with the patient until a friend or family member has arrived. Other information, such as medication use (Figure 4), can be updated if required using a questionnaire.

Having gathered and updated the patient’s mental and physical health status, the intervention continues with multimedia resources that explain aspects of the patient’s symptoms, behaviors, and ways to improve these symptoms. In a series of videos specifically designed for this intervention, an actor explains the symptoms of depression, the vicious circle leading to a worsening of the depression and ways to counteract this vicious circle, the importance of doing pleasant activities to improve mood, how avoidant behaviors are linked to depression, how to plan pleasant (healthy) activities, and how to prevent relapse. As CHWs have limited training in the symptoms of depression and the principles underlying the intervention, these videos support the CHWs in providing patients with the required information on their depressive symptoms and the purpose of the intervention. One of the main goals of the intervention is to strengthen the autonomy of the patients and highlight the role they have in their own improvement. For this reason, in all sessions the patients do activities on the app, supported by the CHW, and plan activities to do between sessions. This strategy helps patients to develop the autonomy to identify and deal with symptoms of depression at present and in the future.

Sessions conclude with the scheduling of a new appointment. This scheduler recommends the date for the next appointment based on the patient’s current progress in the therapy and structures the process of making an appointment for a new visit to complete the current session or to move on to the next session in the therapy. As internet connectivity is not always available at the patients’ homes, the app contains all information for the patients under the care of the CHW who uses the device.

As mentioned previously, an important goal of the system is to empower CHWs with limited training in mental health service provision to provide a psychosocial intervention. To this end, the system provides automated support to the CHWs. This support consists of session content recommendations, as well as support in scheduling intervention visits and further recommendations personalized to that patient, such as warnings regarding adherence to the intervention, suicidal ideation, and progress in the intervention. The CHW has access to these recommendations and warnings in a dedicated screen in the app. Where necessary, the system forwards recommendations and warnings to supervisors by email.

To further support the supervision of CHWs, he or she can, in consultation with the patient, choose to make audio recordings of (parts of) the session for use in future intervention or supervision sessions. These audio recordings are stored on the device and are only accessible to the CHW.

#### Web Interfaces for Monitoring and Supervision

The Web interfaces allow individuals with supervisory roles access to the system. The information shown to these individuals varies slightly based on the objective to show only the most pertinent information. Those that are responsible for the running of the intervention at a high level (Trial Managers or their equivalent in the health system) see aggregate data on a per-patient basis, whereas clinical supervisors see more detailed information for each patient related to the clinical care in which one of their CHWs is involved.

### Experimental Validation and User Experience Testing

The PROACTIVE system was tested in a pragmatic 2-arm pilot study in 2 family health units in the Northern area of São Paulo city, Brazil [41]. A previous survey of older inhabitants of São Paulo’s poor neighborhoods found that only 12.3% of those identified as having depression were receiving treatment [42]. The insufficient availability of mental health services to such populations has previously raised the alarm [43], and PROACTIVE is a first attempt to provide a simple, feasible, and affordable depression intervention to older inhabitants of São Paulo’s poor neighborhoods. Eligible participants were aged 60 years and older. Exclusion criteria were not having depression as assessed by the PHQ-9 (PHQ-9<10); complete deafness; terminal illness; high risk of suicide; or incapability to communicate (for example, cognitive impairment and mental illness either relayed by a family member or detected by the researcher).

Before the start of the intervention, 8 CHWs and 3 nurse assistants (NAs) were trained and provided with an overview of the intervention, session contents, how to use the technological support platform, psychosocial techniques to deliver the intervention, and ways to engage with patients.

The training program consisted of 3 full days of intensive training delivered by 2 psychologists. During the intervention, the system was used for a total of 17 weeks by the 8 CHWs and 3 NAs who cared for a total of 33 patients with 15 individuals in a low-intensity (8 sessions) and 18 in a high-intensity group (11 sessions). Overall, 19 patients completed all sessions within the timeframe available. Completion rate in the low-intensity group was 13 out of 15 with completion in the high-intensity group 4 out of 18. The main reason for the low completion rate in the high-intensity group was the slightly curtailed time available for the follow-up in the pilot study. The mean PHQ-9 at follow-up (approximately 24 weeks after assignment to a CHW) was 12.3 (SD 3.7) and 3.8 (SD 3.9) in the control and intervention arms, respectively, and the follow-up rate was 92% (23/25) and 94% (31/33) in the control and intervention arms, respectively.

Upon completion of the pilot, the 8 CHWs and 2 of the 3 NAs involved in the intervention participated in a qualitative assessment of the system in which the System Usability Scale (SUS) [44] and the Technology Acceptance Model (TAM) [45] were used to elicit the CHW’s perception of usability of the system. The NAs were excluded from this analysis, as their higher levels of training would result in optimistically skewed conclusions. The average age of the CHWs was 37.3 years (SD 6.7 years). Out of the 8 CHWs, 7 indicated they had smartphones and of those with smartphones, all used WhatsApp, 5 used Facebook, and 3 used their smartphones to play games. Half of the respondents had used tablets before their use of tablets in this study. They rated their level of difficulty with the use of technology on average as 1.9 on the scale provided in Multimedia Appendix 1, indicating that, on average, the respondents perceived their use of technology between *A lot of difficulty* and *Cannot handle technology*.

The questions and Portuguese translations of the SUS are shown in Multimedia Appendix 1, with responses provided on a 5-point Likert scale ranging from 0 to 4 with only the end points labelled as strongly disagree (coded as 0) and strongly agree (coded as 4).

The questions and Portuguese translations of the TAM questionnaire can be found in Multimedia Appendix 1. The responses to the TAM questionnaire are provided on a 7-point Likert scale ranging from extremely unlikely (coded as 1) to extremely likely (coded as 7) (see Multimedia Appendix 1). 

For the analysis of results, we calculated the SUS score in the usual way, which involves summing the scores on all positive elements of the questionnaire (ie, all odd-numbered statements), summing the scores on all negative elements (even-numbered statements), and subtracting the latter sum from the first. By adding 20 to the result and subsequently multiplying the total by 2.5, the overall score will be on a scale of 0 to 100. Rather than interpreting the score as a percentage score, it should be seen as a percentile rank with an SUS score of 68 as the mean score [46].

Results from the TAM questionnaire were analyzed as the *perceived usefulness (PU)*, calculated as the mean score of questions 1 to 6 of the TAM questionnaire, and *ease of use (EoU)*, calculated as the mean score of the questions 7 to 12 of the TAM questionnaire.

Written informed consent was received from all participants in the study. The Ethical Committees of the Faculty of Medicine of the University of São Paulo (number 1.339.865) and of the Municipal Health Secretariat of São Paulo (number 1.340.790) approved this study. The trial was registered with the Registro Brasileiro de Ensaios Clínico (ReBEC), number RBR-5nf6wd-ReBEC.

## Results

The mean overall SUS score was 65.6, which in previous studies has been associated with an overall rating of between *OK* and *Good* [47]. Mean ratings of the responses to the individual items are shown in Table 1. These show that, whereas agreement with the positive aspects of the SUS (the odd-numbered questions) is always higher than neutral (a score of 2), agreement with the negative aspects of the SUS questionnaire (the even-numbered items) is higher than neutral for questions 4 and 6. From the questionnaire wording, this indicated that users experienced aspects of the system as inconsistent and expected to need assistance for further use of the system.

The mean scores for *PU* and *EoU* were 5.4 (SD 1.7) and 5.6 (SD 1.3), respectively, which indicates mildly positive views between *somewhat likely* and *likely* on *PU* and *EoU*. Per-question average responses on the *PU* and *EoU* are shown in Table 2 and provide a better insight in aspects of the system that require improvements. The 2 aspects of PU that were scored lowest (PU4 and PU5) indicated that the respondents perceived the ability of PROACTIVE to help them be more effective at doing their job and making the job easier *somewhat likely*. Of note for *PU* is that the participants performed tasks with the app they had never performed before, and their frame of reference did not allow comparison with a past experience without the use of the app. In that respect, the *EoU* responses are of greater interest as they give an insight in usability experiences.

**Table 1:** Mean scores on individual items of the System Usability Scale.

System Usability Scale question	Mean score on question
1	3.1
2	1.4
3	2.6
4	2.5
5	3.5
6	2.1
7	2.6
8	1
9	3.1
10	1.8

**Table 2:** Average scores on the Technology Acceptance Model.

Technology Acceptance Model question	Mean score on question
1	6
2	5.6
3	5.4
4	5
5	5.1
6	5.9
7	5.4
8	5.1
9	6
10	5.9
11	5.1
12	6

In all, 3 items on this scale received mean scores below the overall mean EoU score of 5.6. These items relate to learning to operate (EoU1), using correctly (EoU2), and becoming skillful (EoU5) at use of the app.

**Figure 5:**
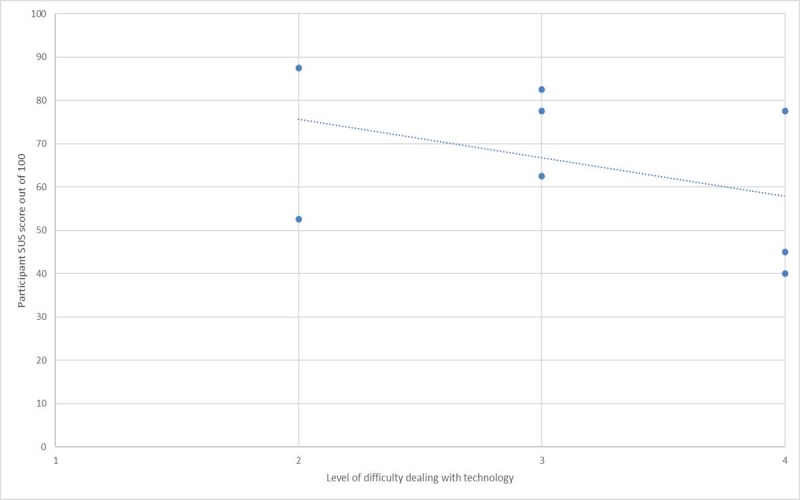
System Usability Scale (SUS) versus technology experience.

**Figure 6:**
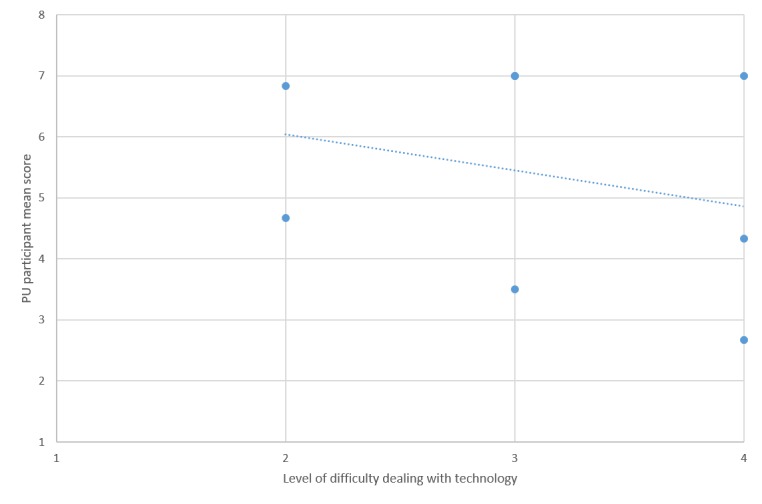
Perceived usefulness (PU) versus technology experience.

**Figure 7:**
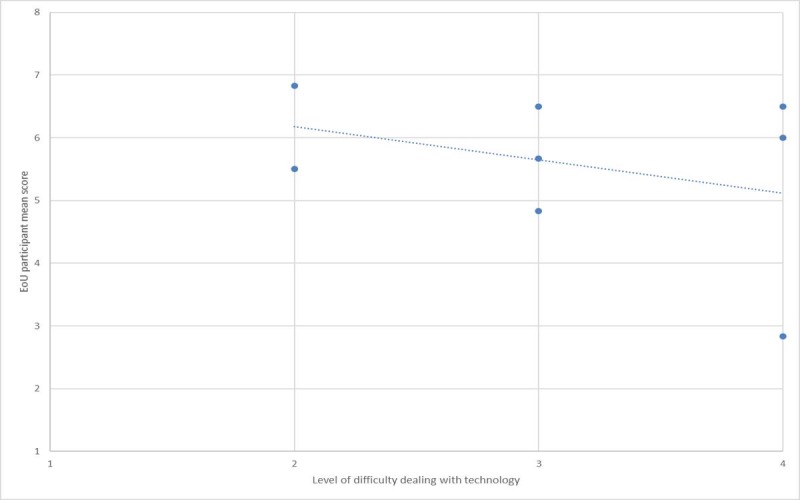
Ease of use (EoU) versus technology experience.

To put this in perspective, Figures 5-7 show plots of the relationships between the user-reported difficulty in using technology versus SUS, PU, and *EoU,* respectively. These figures suggest that lower scores for SUS, PU, and EoU can partly be explained by the participants’ level of difficulty with use of technology. This in turn suggests that the *PU* and *EoU* improvements may be realized through a combination of modifications to the app and the ability to gain more experience in its use. It is acknowledged that these results were obtained in a pilot study from a group of only 8 participants, thus limiting their statistical value.

## Discussion

### Principal Findings

Feedback obtained during the pilot and the qualitative software validation performed with end users provided important insights in the extent to which the high-level requirements identified for the system were implemented successfully.

#### Requirement 1: Guidance

In the usability assessment, some of the users indicated that they found the system inconsistent and expected to require technical assistance during normal use of the system. Whereas these results may partly stem from bugs encountered and resolved during the pilot, they do indicate that further guidance is required. Solutions may be to hide part of the interface, such as the list of pages in a chapter displayed on the left-hand side of the screen by default and more extensive logging of use of the app, such that issues can be identified automatically. For example, longer than expected or usual activity on a particular screen of the app may indicate that users encountered issues with the content presented on the screen and such findings can be discussed in supervision meetings.

#### Requirement 2: Decision Support

Decision support was visible to end users in the form of recommendations on the course of action after 3 initial sessions resulting in the patient being allocated to a high-intensity (8 more sessions) or low-intensity (5 more sessions) intervention, questionnaires on suicidality, and advice to discuss patients that did not show progress (based on trends in PHQ-9 responses) with supervisors. The algorithm for allocation to the low-intensity or high-intensity treatment was not disclosed to the health workers, so they could not influence the allocation of patients to the second phase of treatment.

#### Requirement 3: Supervision

In a full-scale trial or clinical roll-out of this intervention, CHWs will be supervised by the clinical supervisors who normally have supervisory responsibilities for these CHWs. During the pilot, supervision was performed by research psychologists who conducted the pilot study. Data captured by the system were used to discuss the progress of patients and to make suggestions for future sessions.

The functionality to make audio recordings of sessions was added to the app during the pilot upon request. For simplicity and ethical considerations, it was decided not to send the resulting audio files to the server. Hence, reviews can only be done face-to-face with the CHW, who is the only person with access to the files created on the device. It would be worth considering whether the benefits that could be derived from making the audio files available on the server would weigh up against potential issues around privacy. It may also be possible to implement a consent mechanism for audio uploads to the server on a per-user or per-instance basis. A further consideration is that the storage of audio data would require more server storage capacity, but this should not be a limiting factor with modern servers.

#### Requirement 4: Accountability

Accountability functionality was enabled by the patient data gathered during intervention sessions, but also by logs relating to session timing and appointments. The app logged when a session was started and finished and kept track of appointments made with the patient. It also allowed the CHW to make note of missed appointments and the reasons. These data proved invaluable during the pilot in several ways. For example, the patient responses gathered during sessions allowed automated content selection during interventions and the generation of patient-specific prompts to CHWs, thus providing robust support to the latter in following protocols and procedures. The data on scheduled, completed, and missed sessions provided valuable information on patient progress and potential barriers to their involvement in the intervention. For (trial) supervisors, the logged data were presented in such a way that these individuals obtained a high-level overview of the intervention delivered by CHWs and progress of their patients, thus allowing timely corrective action when required.

During the pilot, the geolocation of a session was not recorded and CHWs were also not prevented from accessing a session for a patient when there was no future appointment for that patient. Although this may be seen as overly rigid, there may be merit in locking the app for a user until shortly before the next scheduled appointment. This would prevent inadvertent data logging for a user (for example, because of briefly accessing the next session in the app when merely intending to review the session the next patient will receive) and would also encourage CHWs to maintain complete diaries of patient visits.

Treatment recommendations and warnings regarding a lack of progression or suicidality were displayed both in the app and in the supervisors’ Web interface. For full accountability, a protocol should be implemented that requires CHWs and their supervisors to indicate that the recommendations and warnings have been reviewed and followed up.

#### Requirement 5: Record Keeping

The patient data logged in the sessions were used to personalize subsequent sessions, thus facilitating CHWs to provide a contextualized intervention. This, in turn, enabled sessions to be conducted efficiently (for example, the medical questionnaire can be reviewed and updated very quickly) and allowed an objective review of progress (through reviewing homework agreed on in a previous session and by allowing read-only access to tabular and graphical representations of PHQ-9 response, mood ratings, and homework completion on a session basis). In addition, aggregate information derived from the data gathered during sessions provided supervisors a good insight in patient progress and allowed for timely intervention where required.

Although, where possible, data input was structured and made quicker using drop-down lists and other multiple-choice widgets, some elements of the intervention required considerable free-text input. We provided keyboards for this purpose and though this may suit some users, others may find such input difficult on a tablet device.

#### Requirement 6: Training Environment

Although generally positive, the responses to items 3 and 7 on the SUS scale regarding EoU and learning rate, respectively, indicate that users were not always confident in using the system. Requests for an interface with *dummy* patients for each of the sessions (which was implemented during the pilot) support our belief that these shortcomings can be remedied with improved in-app training facilities.

#### Requirement 7: Automated Communication

During the pilot, automated communication was limited to patient-specific warnings being shown in the Web interfaces for supervisors and researchers. Such automated communications should be extended to automated emails to supervisors and, where necessary, to routine care health providers involved in the care of patients that make use of the system to fully comply with accountability requirements.

#### Requirement 8: User-Initiated Communication

Due to time constraints, user-initiated communications were not implemented during the pilot study, but CHWs were able to contact supervisors using the device’s normal phone app and Google Hangouts. A WhatsApp group was used frequently for communication among CHWs and researchers involved in the trial. The limited implementation of this user-initiated communication in the pilot study version of the system does not seem to have had a negative effect on the good relationship between health care workers in a team, but this is likely because of the fact that these health care teams were already well established.

#### Requirement 9: Delivery in the Community

Mobile delivery of the intervention in patients’ homes was facilitated using the mobile intervention interface on Android tablets that contained data for all the patients under the care of a given CHW using the device. Most tablets were shared by, normally, 2 CHWs, and the protocols required users to synchronize their devices before leaving the community health center. In a few instances, this sharing resulted in CHWs using a different tablet that did not contain the data for their patients. A login in the app with subsequent synchronization would have resulted in all data being downloaded, but this was not always performed, on occasion resulting in CHWs not being able to perform the session as planned. Although the tablets were equipped with subscriber identification module cards, a connection to the system’s servers may not always be available at a patient’s home. For this reason, it is desirable to implement a procedure within the app that unobtrusively ensures the data for the CHW using the tablet are indeed available on the device.

Task-shifting has been the preferred strategy to address the lack of specialized resources in LMICs. However, this problem is of such magnitude that even transferring responsibilities to low-cadre health workers is not enough to overcome the problem. More innovative solutions are needed. One such solution is for technology to provide assistance to these low-cadre health workers. This is already happening in many parts of the world, and the uptake is increasing rapidly [48]. Our pilot suggests that technology indeed may have a positive effect on the ability of low-cadre health workers to effectively deliver a psychosocial mental health intervention. Although our results lack statistical power, they suggest the intervention improved symptoms of depression and that the support provided to the CHWs by the system allowed them to deliver the intervention with very little training and support. Moreover, the pilot addresses the needs of a significantly underserved population, which does not have access to routine mental health services delivered by highly skilled practitioners. The combination of task-shifting and technology is a key enabler for provision of mental health services to this underserved population.

### Limitations

The timelines and resources available for the development of the system were considerably challenging and for this reason, choices had to be made in regard to the implementation of the requirements identified in the Methods section. Shortcomings in this regard were identified in the previous section and form the starting point for further development.

A further limitation related to the usability testing of our system concerns the limited number of users who provided their feedback (8 of the 11 users during the pilot). These numbers do not allow for a robust quantitative analysis of results obtained from the SUS and TAM questionnaires. Nevertheless, the qualitative analysis of their responses has provided invaluable feedback for further development of the system.

As a last limitation, as the supervisor role in the pilot was covered by psychologists involved in the development of the project, we have not yet robustly assessed their experiences with and opinion of the system. This will be rectified during the main trial.

### Conclusions

In this paper, we proposed a set of high-level software requirements for ICT systems supporting task-shifted interventions based on the recommendation framework proposed by Campbell and Scott [8]. These software requirements were used to develop an ICT system for the support of a depression intervention provided by CHWs without specialized mental health training. The system consists of an app used by CHWs to provide a structured intervention to older adults in São Paulo’s (Brazil) poorer neighborhoods and Web interfaces that allow the various stakeholders to monitor and supervise the intervention remotely. The intervention app was built using a purpose-built API that allows direct interaction between the graphical user interface and an underlying data source. The advantage of this approach is that new functionality can be rapidly added by defining the user interface alone. As a result, future development can be performed by users with limited experience in app development.

The SUS and TAM were used to elicit information on usability, PU, and EoU. Results from these assessments and insights gained during the pilot were used to assess the high-level software requirements we proposed. Our overall conclusion is that these high-level requirements adequately captured the functionality required to enable the CHWs to provide the intervention successfully. Nevertheless, the analysis of results indicated that some improvements are required for the system to be useable in a task-shifted intervention. The most important of these are more extensive access to a training environment, access for supervisors to metadata such as duration of sessions or exercises to identify issues, and a more robust and human-error–proof approach to availability of patient data on the mobile devices used during the intervention. Given the rapidly increasing number of task-shifted interventions in health care, which are delivered mostly by CHWs in LMICs, and other nonspecialized health workers in high-income countries, ICT solutions are a promising avenue to provide the support and accountability required in the performance of their demanding tasks.

## Appendix

Multimedia Appendix 1 Questionnaires used in the qualitative study.

## Abbreviations

API: application programming interface
CHW: community health worker
EoU: ease of use
ICT: information and communications technology
LMICs: low-middle-income countries
NAs: nurse assistants
PHQ-9: 9-item Patient Health Questionnaire
PU: perceived usefulness
SUS: System Usability Scale
TAM: Technology Acceptance Model
