# Supplementary material for: Investigating Software Requirements for Systems Supporting Task-Shifted Interventions: Usability Study
Source: J Med Internet Res. 2019 Nov 12;21(11):e11346. doi: 10.2196/11346 (PMC6880237; doi:10.2196/11346)
Supplement: Multimedia Appendix 1 [file jmir_v21i11e11346_app1.pdf]

Appendix 1. Questionnaires used in the qualitative study

Table 1: Description of technology aptitude on 4-point scale

Point value		
1	Can't handle technology	Não sei mexer em aparelhos eletrônicos
2	A lot of difficulty	Tenho muita dificuldade para mexer em aparelhos eletrônicos
3	Some difficulty	Tenho um pouco de dificuldade para mexer em aparelhos eletrônicos
4	No difficulty	Não tenho nenhuma dificuldade para mexer em aparelhos eletrônicos

Table 2: Questions used for the System Usability Scale questionnaire

1	I think that I would like to use this system frequently	Eu gostaria de usar este aplicativo com frequência.
2	I found the system unnecessarily complex	Eu achei o aplicativo mais complexo do que o necessário.
3	I thought the system was easy to use	Eu achei que o aplicativo era fácil de usar.
4	I think that I would need the support of a technical person to be able to use this system	Eu precisei de apoio de outra pessoa para usar o aplicativo.
5	I found the various functions in this system were well integrated	Achei que funções do aplicativo estavam bem integradas.
6	I thought there was too much inconsistency in this system	Eu acho que havia muita inconsistência no aplicativo (o aplicativo parava com frequência).
7	I would imagine that most people would learn to use this system very quickly	Eu imagino que a maioria das pessoas aprenderia a usar o aplicativo muito rapidamente.
8	I found the system very cumbersome to use	Achei o aplicativo muito complicado de usar.
9	I felt very confident using the system	Eu me senti muito confiante usando o aplicativo.

10	I needed to learn a lot of things before I could get going with this system	Eu preciso aprender um monte de coisas antes de poder usar o aplicativo.

Table 3: Questions used for the Technology Acceptance Model questionnaire

1	Perceived Usefulness	Using PROACTIVE in my job would enable me to accomplish tasks more quickly.	Usar o PROACTIVE no meu trabalho me permitiu realizar tarefas mais rapidamente.
2		Using PROACTIVE would improve my job performance.	Usar o PROACTIVE melhorou meu desempenho no trabalho.
3		Using PROACTIVE in my job would increase my productivity.	Usar o PROACTIVE no meu trabalho aumentou minha produtividade.
4		Using PROACTIVE would enhance my effectiveness on the job.	Usar o PROACTIVE aumentou minha eficácia no trabalho.
5		Using PROACTIVE would make it easier to do my job.	Usar o PROACTIVE tornou meu trabalho mais fácil.
6		I would find PROACTIVE useful in my job.	Eu achei que o PROACTIVE foi útil no meu trabalho.
7	Ease of Use	Learning to operate PROACTIVE would be easy for me.	Aprender a mexer no PROACTIVE foi fácil para mim.
8		I would find it easy to get PROACTIVE to do what I want it to do.	Quando precisei fazer algo no PROACTIVE, consegui fazer sem problemas.
9		My interaction with PROACTIVE would be clear and understandable.	Minha interação com o PROACTIVE foi clara e compreensível.
10		I would find PROACTIVE to be flexible to interact with.	Eu achei o PROACTIVE flexível para interagir.
11		It would be easy for me to become skillful at using PROACTIVE.	Foi fácil adquirir habilidade para usar o PROACTIVE.

12		I would find PROACTIVE easy to use.	Eu achei o PROACTIVE fácil de usar.

Table 4: Response options used for the TAM questionnaire

1	Extremely Unlikely	Extremamente improvável
2	Unlikely	Improvável
3	Somewhat Unlikely	Um pouco improvável
4	Neutral	Neutro
5	Somewhat Likely	Um pouco provável
6	Likely	Provável
7	Extremely Likely	Extremamante provável
